# Prevalence and Determinants of Tobacco Use in India: Evidence from Recent Global Adult Tobacco Survey Data

**DOI:** 10.1371/journal.pone.0114073

**Published:** 2014-12-04

**Authors:** Akansha Singh, Laishram Ladusingh

**Affiliations:** 1 International Institute for Population Sciences, Mumbai, India; 2 Department of Mathematical Demography and Statistics, International Institute for Population Sciences, Mumbai, India; Geisel School of Medicine at Dartmouth College, United States of America

## Abstract

**Background:**

Tobacco use in India is characterized by a high prevalence of smoking and smokeless tobacco use, with dual use also contributing a noticeable proportion. In the context of such a high burden of tobacco use, this study examines the regional variations, and socioeconomic, demographic and other correlates of smoking, smokeless tobacco and dual use of tobacco in India.

**Methods and Findings:**

We analyzed a cross sectional, nationally representative sample of individuals from the Global Adult Tobacco Survey in India (2009–10), which covered 69,296 individuals aged 15 years and above. The current tobacco use in three forms, namely, smoking only, smokeless tobacco use only, and both smoking and smokeless tobacco use were considered as outcomes in this study. Descriptive statistics, cross tabulations and multinomial logistic regression analysis were adopted as analytical tools. Smokeless tobacco use was the major form of tobacco use in India followed by smoking and dual tobacco use. Tobacco use was higher among males, the less educated, the poor, and the rural population in India. Respondents lacking knowledge of health hazards of tobacco had higher prevalence of tobacco use in each form. The prevalence of different forms of tobacco use varies significantly by states. The prevalence of tobacco use increases concomitantly with age among females. Middle-aged adult males had higher prevalence of tobacco use. Age, education and region were found to be significant determinants of all forms of tobacco use. Adults from the poor household had significantly higher risk of consuming smokeless tobacco. Lack of awareness about the selected hazards of tobacco significantly affects tobacco use.

**Conclusions:**

There is an urgent need to curb the use of tobacco among the sub-groups of population with higher prevalence. Tobacco control policies in India should adopt a targeted, population-based approach to control and reduce tobacco consumption in the country.

## Introduction

Tobacco is used in India in many forms. Smoking of cigarettes and *beedis* (tobacco wrapped in dried leaves of special trees) is one form of tobacco use. Smokeless tobacco use consists of chewing *pan* (mixture of lime, pieces of areca nut, tobacco and spices wrapped in betel leaf), chewing *gutkha* or *pan masala* (scented tobacco mixed with lime and areca nut, in powder form), and *mishri* (a kind of toothpaste used for rubbing on gums). India has one of the highest tobacco users in the world both in number and relative share. India is one of the fewer countries in the world where prevalence of smoking and smokeless tobacco use are high and is characterised by dual use of tobacco (use of both smoking and smokeless tobacco products) also contributes to a noticeable proportion. Using data from the National Family Health Survey second round (NFHS II, 1998–99), prevalence of tobacco use in India was estimated to be 37 percent among the population of 15 years and above [1]. High prevalence of tobacco use in any form is also reported among school going adolescents aged 13–15 years [2]. This high burden of tobacco use is associated with a high mortality burden. According to the World Health Organization (WHO), nearly 6 million deaths occur every year due to tobacco use, which may escalate to 8 million deaths a year by 2030 [3]. Smoking is responsible for a large number of premature deaths in India. The majority of smoking related deaths in India occur in the prime working age group of 15–59 years [4]. Smokeless tobacco use is also associated with the increasing risk of cancer. Smokeless tobacco is also highly addictive and causes cancer of the head and neck, oesophagus and pancreas, besides many oral diseases [3].

Establishment of effective tobacco control policies depends on precisely assessing the enormity of tobacco use in the country. Several national level studies have collected information on tobacco use to evaluate the prevalence of tobacco use. Tobacco use is a part of the consumer behaviour component of the National Sample Survey (NSS), held every five years [5]. The 52^nd^ round of the NSS on Morbidity and Private Health Expenditure, conducted between June 1995 and June 1996 by the National Sample Survey Organization (NSSO) of the Government of India, collected information on current regular smoking and chewing of tobacco for all individuals aged 10 years and above. The survey estimated tobacco use prevalence among males to be at 51.3% and among females at 10.3% [6]. Another nationwide survey, the National Family Health Survey (NFHS), in its second and third rounds, collected some data on tobacco use in India. The second round collected information on tobacco use for the household members aged 15 years and above, and estimated that the prevalence among males was 46.5% and that among females was 13.8% [7]. The third round, held in 2005–2006, collected data on tobacco use from a sample of 124,385 females aged 15–49 years and 74,369 males aged 15–54 years from all 29 states in India. The survey estimated that the prevalence of tobacco use among males was 57.6% and among females 10.8% [8]. The National Household Survey of Drug and Alcohol Abuse in India (NHSDAA), conducted in 2002 among males, covered over 40,000 individuals aged 12–60 years in nearly 20,000 households in 25 states. The overall prevalence of current tobacco use from the NHSDAA was found to be 55.8% [9]. The recently conducted Global Youth Tobacco Survey in 2006 and 2009 in India includes data on cigarette and other forms of tobacco use as well as information on five determinants of tobacco use among adults aged 13–15 years. The survey estimated that the prevalence of current tobacco use increased from 13.7% in 2006 to 14.6% in 2009 [10].

All these studies were mostly limited to collecting some data on tobacco use as a component of health-based surveys. However, there was a lack of a large scale survey with comprehensive data on tobacco use among adults in India. Most of the past research work on tobacco was either based on localized studies with an urban bias or based on non-representative sample surveys with socio-demographic predictors of tobacco related behaviour mostly inadequately understood. These works were frequently limited to specific focus group in terms of their tobacco habit.

The recent two rounds of the Global Youth Tobacco Survey were specially designed to collect tobacco related information, although for a very specific age group. The Global Adult Tobacco Survey (GATS) is a standardized household survey that enables countries to compile data on key tobacco indicators and assist states in the formulation, tracking and execution of effective tobacco control interventions and international comparisons as laid out in the MPOWER policy package of the WHO [11]. The GATS-India survey is the first large scale survey designed to collect reliable information on tobacco use in the country among adults aged 15 years and above. This survey gives us an ample opportunity to look into the current burden of tobacco use among the adult population aged 15 years and above in India, and it’s associated socioeconomic, demographic and knowledge related factors. GATS-India is the first large scale survey conducted after the enactment of the Cigarettes and Other Tobacco Products Act (COTPA) (2003) and the launch of the National Tobacco Control Programme (2007–08) in India. This study serves the purpose of assessment of national programmes for the control of tobacco use mentioned in another study [12]. The present study is an attempt to examine the regional variations in the tobacco use in India and to study the socioeconomic, demographic and knowledge related correlates of tobacco use in the form of smoking only, smokeless tobacco only and dual use.

## Methods

### Data

This study is based on the secondary analysis of the Global Adult Tobacco Survey India (GATS-India) 2009–10 data. This survey data was released for the general researchers by International Institute for Population Sciences. Global Adult Tobacco Survey is the global standard for systematically monitoring adult tobacco use (smoking and smokeless) and tracking key tobacco control indicators. GATS-India is conducted globally in around 14 countries. The Centers for Disease Control and Prevention (CDC), CDC Foundation, Johns Hopkins Bloomberg School of Public Health (JHSPH), Research Triangle Institute International (RTI International), the World Health Organization and many countries throughout the world worked together to design and implement GATS. For each participating country, a standard protocol with respect to questionnaire, sample design, data collection and management procedures was used. Survey information was collected using handheld devices. The Ministry of Health and Family Welfare, Government of India, designated the International Institute for Population Sciences, as the nodal agency for conducting GATS Survey in India. The main objectives of the GATS India Survey were to measure the impact of tobacco control efforts through implementation of different provisions of COTPA 2003 and its regulations and to systematically monitor adult tobacco use and track key tobacco control indicators. Further information regarding the guidelines followed to collect the data is available in the GATS-India report [11].

GATS-India (2009–10) is a nationally representative household survey covering population aged 15 years and above, covering all the 29 states and 2 Union Territories (UTs) in India. Multistage sampling procedure was adopted independently in each state, and within the states, independently in urban and rural areas to select the sample. In the urban areas, three-stage sampling was adopted for the selection of households. At the first stage, a list of wards from all cities and towns of the state/UT formed the urban sampling frame from which a required sample of wards, i.e., primary sampling units (PSUs), was selected using probability proportional to size (PPS) sampling. At the second stage, a list of census enumeration blocks (CEBs) in every selected ward formed the sampling frame from which one CEB was selected by PPS from each selected ward. At the third stage, a list of all the residential households in each selected CEB formed the sampling frame from which a sample of the required number of households was selected. In the rural areas, two-stage sampling was adopted for the selection of households. The PSUs were villages selected using the PPS sampling method. At the second stage, a list of all the residential households in each selected village formed the sampling frame from which a sample of the required number of households was selected. From each eligible household, one respondent was selected.

Complete data is available for 69,296 adult respondents age 15 and above, of which 33,767 and 35,529 were males and females respectively. The survey covered domains like tobacco use (smoking and smokeless tobacco), exposure to second hand smoke, cessation, economics of tobacco, exposure to media messages on tobacco use, and knowledge, attitudes and perceptions towards tobacco use. The survey was designed to provide estimates of the tobacco prevalence at the national and the state levels, and by certain specific background characteristics. Data for tobacco use was collected from the eligible respondents aged 15 years and above. The respondents were asked about their daily and occasional use of tobacco. Smoking tobacco included *beedi*s, cigarettes, cigars, cheroots, rolled cigarettes, tobacco rolled in maize leaf and newspaper, hookah, pipes, *chillum*, and *chutta*. Smokeless tobacco included tobacco leaf, betel quid with tobacco, *sada/surti*, khaini or tobacco lime mixture, *gutkha*, *pan masala* with *zarda, gul, gudaku, and mishri*. Final sample included in the analysis were all the 69,296 respondents [11].

### Outcome variable

The main dependant variable in the analysis is the tobacco use categorized into four types, namely, smoking only, smokeless tobacco use only, dual use of tobacco (both smoking and smokeless tobacco use) and non-user of tobacco.

### Independent variables

The state level variations in type of tobacco use were assessed for the 29 states and 2 UTs of India. The relationship of age with use of tobacco in three forms was assessed by classifying age into age groups of five years. For the further analysis, independent variables used were age (four categories), sex (male/female), residence (rural/urban), education (four categories), occupation (four categories), region (six categories), knowledge that exposure to smoking causes heart attack, stroke and lung cancer (Yes/No), smokeless tobacco causes serious illness (Yes/No), and household asset scores (three categories). Six geographical regions, covering 29 states and 2 UTs, were included in the analysis. Included in the North were the states of Jammu & Kashmir, Himachal Pradesh, Punjab, Chandigarh, Uttarakhand, Haryana and Delhi; in the Central region, Rajasthan, Uttar Pradesh, Chhattisgarh and Madhya Pradesh were included; the East included West Bengal, Jharkhand, Odisha and Bihar; the North-east included Sikkim, Arunachal Pradesh, Nagaland, Manipur, Mizoram, Tripura, Meghalaya and Assam; in the West were included Gujarat, Maharashtra and Goa; and finally Andhra Pradesh, Karnataka, Kerala, Tamil Nadu and Puducherry were included in the South region. The household economic status was assessed using the household assets information provided. Since the information of assets was quite limited in nature, the scores of 10 household assets were summed up to give a final score between 0 and 10. These scores were divided into three parts based on their distribution, and households were categorised as poor, moderate and rich.

### Statistical analysis

The prevalence of tobacco use for each type namely, smoking only, smokeless tobacco use only, and dual use of tobacco (both smoking and smokeless tobacco use) is defined as the number of persons consuming tobacco in this manner per 100 adult persons 15 years and above. Bivariate analysis was used to estimate the prevalence of tobacco use in three forms by background characteristics. The state level variations in type of tobacco use were assessed by simple bivariate analysis in 29 states and 2 UTs of India. The relationship of age with use of tobacco was assessed by classifying age into age groups of five years. Further prevalence of tobacco use was analysed according to the background characteristics described in the earlier section. Both point estimates and robust 95% confidence intervals were given based on robust standard errors adjusting for strata and clustering at PSU. Multinomial logistic regression is used to estimate and assess the adjusted associations respectively of different socioeconomic, demographic and knowledge related characteristics. Multinomial logistic regression analysis was used to analyze the four outcomes-smoking only, smokeless tobacco use only, dual use of tobacco and non user in a single model. In multinomial regression each outcome is modelled relative to baseline outcome group: non-user of tobacco (in this study). Therefore, relative risk ratios (RRR) are reported rather than risk ratio or odds ratio. The RRR and their 95% confidence intervals were provided in the tables. Models are used after applying the sampling weights and adjusting for multistage sampling designs using svy command in STATA. STATA 11.0 was used to carry out the statistical analysis.

## Results

**Figure 1:**
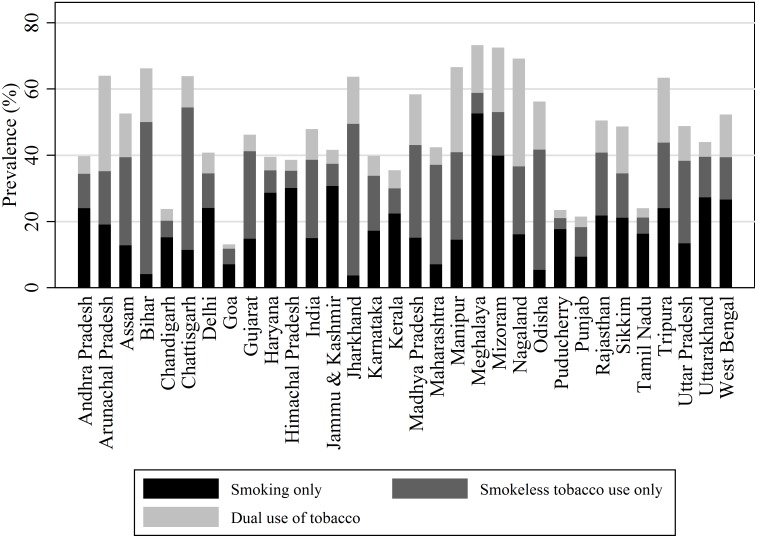
Prevalence of type of tobacco use among males in India by state, GATS India, 2009–10.

**Figure 2:**
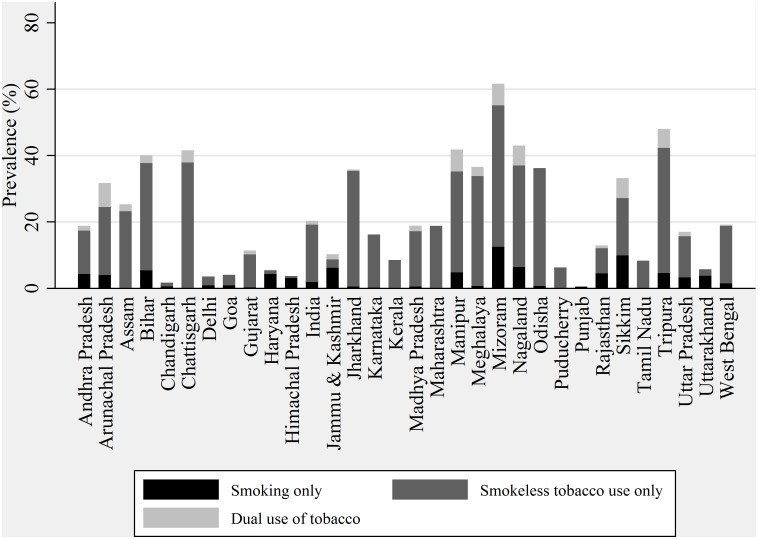
Prevalence of type of tobacco use among females in India by state, GATS India, 2009–10.

Figure 1 and 2 provides the prevalence of tobacco in the form of smoking, smokeless tobacco, and dual use of tobacco (both smoking and smokeless tobacco use) according to sex by states of India. Prevalence of smokeless tobacco use is highest in India followed by smoking and dual use of tobacco both among males and females. In India, tobacco use varies significantly by states. Minimum prevalence of smoking was observed in Goa and maximum in Meghalaya. Similarly, the prevalence of smoking among females varies from as low as 0.0 percent in five states (Chhattisgarh, Maharashtra, Tamil Nadu, Kerala, Puducherry, and Maharashtra) to maximum in Mizoram. Smokeless tobacco use constitutes a major proportion of the overall tobacco use in most of the states. Tobacco use in every form was much higher among males than among females in every state of India. The difference between the maximum and the minimum reported prevalence by state was also significantly higher among males than among females. Among males, Meghalaya had the highest prevalence of smoking, Bihar the highest prevalence of smokeless tobacco use, and Nagaland the highest prevalence of dual tobacco use. Among females, Mizoram has the highest prevalence of smoking and smokeless tobacco use, while Arunachal Pradesh had the highest prevalence of dual use of tobacco. The north-eastern states of India had higher prevalence of every form of tobacco use than the other states. The use of smokeless tobacco was more common in the eastern and central region states of the country.

**Figure 3:**
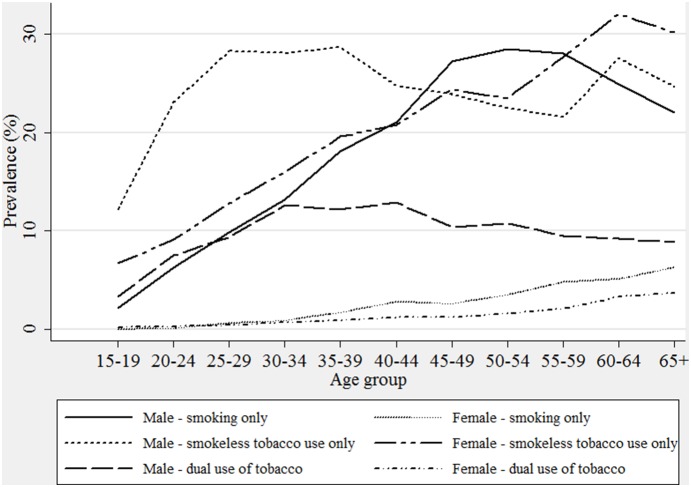
Prevalence of type of tobacco use according to sex and age group, GATS India, 2009–10.

Figure 3 shows the relationship between age and the use of tobacco in India. The prevalence of tobacco use by type is estimated for each five year age group from 15–19 years to 60–64 years, and 65+ as an extensive category for India. The prevalence of smoking increases to the maximum level in the age group 50–54 years, smokeless tobacco in the age group 35–39 years, and that of dual use of tobacco in the age group 40–44 years among males. The prevalence of all forms of tobacco increases linearly with age among females. The sex differentials were higher in the case of prevalence of smoking and dual use of tobacco than that of the use of smokeless tobacco.

Table 1 summarizes the prevalence of smoking only, smokeless tobacco use only, and dual use of tobacco among males in India by different socioeconomic and demographic characteristics. Almost half of the tobacco users in India use smokeless tobacco; this was followed by smoking and dual use of tobacco. The prevalence varies considerably by rural-urban residence, age, household economic status, and knowledge of health hazards of smoking and smokeless tobacco use. The prevalence of smoking was higher among the higher age adults, those living in the rural areas, the uneducated, and the poor as compared to the younger population, those living in the urban areas, the educated, and the rich. Smokeless tobacco use and dual use was highest in the age group 25–44 years. The rich-poor differences were more observed among the smokeless tobacco and dual users. The reported prevalence of different types of tobacco use was much higher among the respondents with no knowledge of the health hazards of smoking and smokeless tobacco use. Prevalence varies considerably by the knowledge of smoking association with lung cancer, and smokeless tobacco health hazards than the other two diseases. The north-east region had the highest prevalence of smoking and dual use of tobacco. Smokeless tobacco use was the highest in the eastern region in India.

**Table 1:** Prevalence of type of tobacco use in India by background characteristics among males, GATS India, 2009–10.

	Smoking only		Smokeless tobacco useonly		Dual use of tobacco	
	%	95%CI	%	95%CI	%	95%CI
**Age group**						
15–24	4.3	[3.5–5.1]	17.7	[15.8–19.6]	5.4	[4.5–6.4]
25–44	15.3	[14.0–16.6]	27.6	[26.2–29.1]	11.7	[10.7–12.7]
45–64	27.3	[25.5–29.2]	23.7	[21.9–25.6]	10.1	[8.9–11.3]
65+	22.1	[19.0–25.1]	24.7	[22.0–27.4]	8.9	[7.3–10.5]
**Residence**						
Urban	14.0	[13.0–14.9]	17.1	[16.0–18.2]	6.4	[5.8–7.1]
Rural	15.5	[14.1–16.8]	26.4	[24.7–28.0]	10.5	[9.6–11.3]
**Education**						
More than secondary	9.0	[8.0–10.0]	15.3	[13.7–16.8]	4.3	[3.6–5.1]
Upto secondary	10.4	[9.4–11.4]	22.0	[20.4–23.6]	7.2	[6.4–8.1]
Upto primary	18.0	[16.1–19.9]	28.0	[26.3–29.7]	12.1	[10.9–13.3]
No education	25.0	[22.9–27.1]	29.1	[26.7–31.4]	13.9	[12.5–15.4]
**Occupation**						
Govt./Non-Govt. employee	16.4	[15.1–17.8]	24.8	[23.1–26.6]	10.5	[9.4–11.5]
Self employed	17.2	[15.8–18.6]	28.0	[26.1–29.8]	10.5	[9.5–11.5]
Homemaker	22.5	[15.7–29.2]	20.0	[12.7–27.3]	13.3	[8.4–18.1]
Others^(a)^	7.4	[6.4–8.4]	13.0	[11.6–14.4]	4.4	[3.6–5.2]
**Household asset**						
Rich	13.5	[12.4–14.6]	13.8	[12.6–14.9]	6.3	[5.4–7.1]
Moderate	15.9	[14.6–17.3]	22.5	[20.9–24.1]	8.4	[7.4–9.3]
Poor	15.4	[13.8–17.0]	31.5	[29.6–33.3]	12.1	[11.0–13.2]
**Smoking causes heart** **attack**						
Yes	13.6	[12.7–14.6]	21.9	[20.7–23.2]	8.3	[7.6–9.0]
No	17.6	[16.2–19.1]	26.8	[24.8–28.7]	11.1	[9.9–12.2]
**Smoking causes stroke**						
Yes	13.1	[12.0–14.1]	22.0	[20.7–23.4]	8.3	[7.5–9.1]
No	17.0	[15.7–18.3]	25.2	[23.6–26.9]	10.3	[9.4–11.2]
**Smoking causes lung** **cancer**						
Yes	13.9	[13.0–14.9]	23.1	[21.9–24.2]	8.9	[8.2–9.5]
No	22.3	[19.9–24.7]	27.4	[24.5–30.2]	12.2	[10.4–13.9]
**Smokeless tobacco use** **causes serious illness**						
Yes	14.1	[13.1–15.0]	23.5	[22.3–24.7]	8.9	[8.3–9.5]
No	23.6	[20.6–26.6]	24.7	[21.8–27.5]	12.7	[10.7–14.7]
**Region**						
North	19.6	[18.1–21.0]	8.4	[7.1–9.7]	3.7	[3.1–4.4]
Central	14.7	[12.8–16.6]	27.7	[25.7–29.7]	11.0	[9.9–12.1]
East	12.7	[10.9–14.6]	31.8	[29.9–33.8]	14.5	[13.0–15.9]
North-east	17.0	[15.6–18.5]	23.8	[21.7–25.9]	16.0	[14.4–17.7]
West	9.7	[8.5–10.8]	28.5	[26.2–30.9]	5.2	[4.2–6.1]
South	20.0	[18.4–21.5]	9.9	[8.5–11.3]	4.8	[3.9–5.7]

Note: CI-Confidence interval; Govt. = Government; ^(a)^Others includes student, retired or unemployed.

Table 2 summarizes the prevalence of smoking only, smokeless tobacco use only, and both smoking and smokeless tobacco use in India by different socioeconomic and demographic characteristics among females. Smokeless tobacco use constitutes almost 90 per cent of tobacco users among females in India, which is much higher as compared to the males. Smoking and dual use of tobacco is prevalent among the uneducated women only. Government and non-government female employees have the highest prevalence of all types of tobacco use. The use of all forms of tobacco increases with increase in age among females. The type of tobacco use varies significantly by economic status and knowledge of health hazards of smoking, and smokeless tobacco. Differences in prevalence of smoking and smokeless tobacco use were observed with regard to the knowledge of smokeless tobacco’s correlation with serious illnesses, and smoking’s correlation with lung cancer and heart attack. This is not, however, the case with the dual users. The north-east region had the highest prevalence of dual use of tobacco. Prevalence of smoking and smokeless tobacco use were highest in the eastern region in India.

**Table 2:** Prevalence of type of tobacco use in India by background characteristics among females, GATS India, 2009–10.

	Smoking only		Smokeless tobaccouse only		Dual use of tobacco	
	%	95%CI	%	95%CI	%	95%CI
**Age group**						
15–24	0.1	[0.0–0.1]	8.0	[7.1–8.9]	0.3	[0.1–0.4]
25–44	1.4	[1.0–1.7]	16.8	[15.5–18.2]	0.8	[0.6–1.0]
45–64	3.8	[3.0–4.6]	26.4	[24.7–28.2]	1.9	[1.4–2.4]
65+	6.3	[4.5–8.1]	30.2	[27.1–33.3]	3.7	[2.3–5.0]
**Residence**						
Urban	0.7	[0.5–1.0]	10.7	[9.8–11.7]	0.4	[0.2–0.6]
Rural	2.3	[1.9–2.8]	20.0	[18.9–21.1]	1.3	[1.1–1.6]
**Education**						
More than secondary	0.1	[0.0–0.2]	3.7	[2.7–4.6]	0.3	[0.0–0.5]
Upto secondary	0.1	[0.0–0.1]	7.1	[6.2–8.0]	0.2	[0.1–0.3]
Upto primary	0.7	[0.3–1.0]	17.8	[16.4–19.3]	0.6	[0.4–0.8]
No education	4.0	[3.3–4.7]	26.7	[25.2–28.2]	2.1	[1.6–2.5]
**Occupation**						
Govt./Non-Govt. employee	2.6	[1.6–3.6]	22.6	[20.6–24.7]	2.5	[1.6–3.3]
Self employed	1.4	[0.9–1.9]	22.9	[20.4–25.4]	0.6	[0.4–0.9]
Homemaker	1.8	[1.4–2.2]	16.3	[15.3–17.3]	1.0	[0.7–1.2]
Others^(a)^	1.7	[1.0–2.4]	10.7	[9.0–12.4]	0.6	[0.2–1.0]
**Household asset**						
Rich	0.7	[0.4–1.0]	7.5	[6.5–8.4]	0.3	[0.2–0.5]
Moderate	1.6	[1.1–2.1]	15.8	[14.3–17.3]	0.9	[0.5–1.2]
Poor	2.9	[2.3–3.5]	25.5	[24.2–26.7]	1.8	[1.4–2.2]
**Smoking causes heart** **attack**						
Yes	1.2	[0.9–1.5]	14.3	[13.4–15.3]	1.0	[0.8–1.3]
No	2.9	[2.3–3.5]	22.4	[20.9–23.8]	1.2	[0.8–1.5]
**Smoking causes stroke**						
Yes	1.3	[1.0–1.7]	14.8	[13.8–15.8]	1.2	[0.9–1.5]
No	2.4	[1.9–2.9]	19.7	[18.3–21.1]	1.0	[0.7–1.2]
**Smoking causes lung cancer**						
Yes	1.6	[1.3–1.9]	15.8	[14.9–16.7]	0.9	[0.7–1.1]
No	3.1	[2.2–3.9]	24.5	[22.3–26.6]	1.9	[1.2–2.6]
**Smokeless tobacco use** **causes serious illness**						
Yes	1.6	[1.3–1.9]	16.3	[15.4–17.3]	1.0	[0.8–1.1]
No	3.7	[2.6–4.7]	24.1	[21.7–26.5]	1.9	[1.1–2.8]
**Region**						
North	2.4	[1.8–2.9]	1.0	[0.7–1.3]	0.3	[0.1–0.6]
Central	2.4	[1.7–3.0]	17.0	[15.3–18.6]	1.7	[1.2–2.2]
East	2.6	[1.8–3.4]	27.6	[25.4–29.8]	1.0	[0.7–1.4]
North-east	1.6	[1.2–2.0]	26.0	[23.1–28.9]	3.2	[2.4–4.0]
West	0.1	[0.0–0.2]	15.6	[13.7–17.4]	0.4	[0.1–0.7]
South	1.5	[0.9–2.2]	11.7	[9.8–13.5]	0.5	[0.1–0.9]

Note: CI-Confidence interval; Govt. = Government; ^(a)^Others includes student, retired or unemployed.

Table 3 gives the results of multinomial logistic regression of type of tobacco use among males. Age was the significant factor associated with all forms of tobacco use among males in India. The highest likelihood of smoking among males was in the age group 45–64 years (RRR: 8.41, 95% CI: 6.85–10.33), that of the use of smokeless tobacco is in the age group 65+ years (RRR: 2.15, 95% CI: 1.75–2.65), and that of use of both types of tobacco was in the age group 25–44 years (RRR: 2.81, 95% CI: 2.29–3.45). Residing in the rural areas increases the likelihood of consuming smokeless tobacco and dual use of tobacco by 1.3 times. The educational status of a person was significantly associated with the use of different forms of tobacco. For instance, the uneducated males had a higher RRR of smoking, consuming smokeless tobacco or both forms of tobacco than those who were educated at the secondary school and higher levels. (RRR: 3.35, 95% CI: 2.81–3.99 for smoking; RRR: 2.10, 95% CI: 1.77–2.48 for smokeless tobacco use; RRR: 4.25, 95% CI: 3.31–5.47 for dual use of tobacco). A comparison of relative risk for all the three forms of tobacco use suggests that the RRR of consuming both smoking and smokeless tobacco is the highest among the uneducated males. As compared to government and non-government employees, others have a significantly lower probability of consuming any form of tobacco. A decrease in the economic status was associated with a higher probability of consuming smokeless tobacco among males (RRR: 1.64 and 1.91 for moderate and poor). Males with no knowledge of smoking association with heart attack had a higher probability of consuming different forms of tobacco. No knowledge of smoking association with lung cancer and of the health hazards of smokeless tobacco increases the RRR of smoking by 1.31 and 1.21 times. Except for the non-awareness of smoking association with heart attack, no other knowledge-related factor had any significant effect on the use of smokeless tobacco or dual use of tobacco. Significant regional differences were observed for smokeless tobacco user or dual use of tobacco. Except for the southern region, males from all the other regions had a higher risk of using smokeless tobacco or dual use of tobacco. Males in the eastern region had the highest RRR of consuming smokeless tobacco (RRR: 4.88, 95% CI: 3.98–5.99), whereas those in the north-eastern region had the highest RRR of smoking (RRR: 1.58, 95% CI: 1.30–1.92) and dual use of tobacco (RRR: 7.26, 95% CI: 5.58–9.43).

**Table 3:** Relative risk ratio (RRR) and their 95%CI estimated from the multinomial regression analysis for the type of tobacco use among males in India, GATS India, 2009–10.

	Smoking only		Smokeless tobacco use only		Dual use of tobacco	
	RRR	95%CI	RRR	95%CI	RRR	95%CI
**Age group**						
15–24®	1.00		1.00		1.00	
25–44	4.41**	[3.65–5.33]	2.08**	[1.75–2.47]	2.81**	[2.29–3.45]
45–64	8.41**	[6.85–10.33]	2.07**	[1.73–2.48]	2.65**	[2.10–3.36]
65+	5.81**	[4.53–7.45]	2.15**	[1.75–2.65]	2.13**	[1.61–2.84]
**Residence**						
Urban®	1.00		1.00		1.00	
Rural	1.09	[0.95–1.25]	1.33**	[1.17–1.51]	1.30**	[1.09–1.54]
**Education**						
More than secondary®	1.00		1.00		1.00	
Upto secondary	1.43**	[1.22–1.67]	1.50**	[1.30–1.72]	1.98**	[1.57–2.50]
Upto primary	2.62**	[2.22–3.10]	2.03**	[1.74–2.37]	3.70**	[2.94–4.66]
No education	3.35**	[2.81–3.99]	2.10**	[1.77–2.48]	4.25**	[3.31–5.47]
**Occupation**						
Govt./Non-Govt. employee®	1.00		1.00		1.00	
Self employed	0.93	[0.82–1.06]	0.94	[0.81–1.08]	0.84*	[0.71–0.99]
Homemaker	0.96	[0.63–1.48]	0.50*	[0.31–0.82]	0.74	[0.45–1.23]
Others^(a)^	0.52**	[0.43–0.63]	0.40**	[0.33–0.49]	0.39**	[0.30–0.50]
**Household asset**						
Rich®	1.00		1.00		1.00	
Moderate	1.17	[1.00–1.37]	1.64**	[1.41–1.90]	1.14	[0.92–1.41]
Poor	1.04	[0.88–1.22]	1.91**	[1.64–2.23]	1.16	[0.94–1.44]
**Smoking causes heart attack**						
Yes®	1.00		1.00		1.00	
No	1.05	[0.92–1.21]	1.06	[0.92–1.24]	1.08	[0.90–1.29]
**Smoking causes stroke**						
Yes®	1.00		1.00		1.00	
No	1.22*	[1.06–1.40]	1.15*	[1.01–1.30]	1.25*	[1.06–1.46]
**Smoking causes lung** **cancer**						
Yes®	1.00		1.00		1.00	
No	1.31**	[1.09–1.57]	1.19	[0.96–1.46]	1.11	[0.86–1.43]
**Smokeless tobacco use** **causes serious illness**						
Yes®	1.00		1.00		1.00	
No	1.21*	[1.00–1.47]	0.93	[0.78–1.11]	1.24	[0.98–1.58]
**Region**						
North®	1.00		1.00		1.00	
Central	1.12	[0.93–1.35]	3.70**	[3.05–4.50]	3.87**	[3.03–4.93]
East	1.07	[0.87–1.32]	4.88**	[3.98–5.99]	5.79**	[4.55–7.37]
North-east	1.58**	[1.30–1.92]	4.17**	[3.26–5.34]	7.26**	[5.58–9.43]
West	0.54**	[0.45–0.64]	3.41**	[2.71–4.30]	1.51**	[1.09–2.09]
South	0.85*	[0.74–0.99]	0.93	[0.72–1.18]	1.03	[0.75–1.41]

Note: CI-Confidence interval; *p<0.05, **p<0.01; ® = Reference category; Govt. = Government; ^(a)^Others includes student, retired or unemployed.

Table 4 summarizes the results of multinomial logistic regression of different types of tobacco use among females by different socioeconomic, demographic and knowledge-related characteristics. Increase in age was significantly associated with a higher probability of using tobacco (smoking, smokeless or both). The RRR of smoking among elderly females, aged 65+ years, was 50.47 times, that of smokeless tobacco use was 4.47 times, and that of dual use of tobacco was 15.20 times higher than among females in the age group 15–24 years. Uneducated females had a higher likelihood of smoking, using smokeless tobacco and dual use of tobacco as compared to those educated up to secondary school and higher levels. Risk of consuming smokeless tobacco was also higher among secondary educated females. Results indicate that self-employed females had a lower risk of consuming smokeless tobacco or dual tobacco use. Living in poor households increases the risk of consuming smokeless tobacco by 2.04 times, and dual use of tobacco by 2.39 times. No knowledge of smoking association with heart attack significantly increases the probability of smoking (RRR: 1.72, 95%CI: 1.07–2.76) or using smokeless tobacco (RRR: 1.22, 95%CI: 1.08–1.39). Non-awareness of smoking association with lung cancer increases the RRR of dual use of tobacco by 2.18 times. Significant regional differences were observed in the consumption of smokeless tobacco use among females. The RRR of consuming smokeless tobacco was 41.45 times higher and that of dual use of tobacco 20.10 times higher among females in the north-eastern region than among females in the northern region. Females in the western region had significantly lower RRR (RRR: 0.06, 95% CI: 0.02–0.16) of smoking than females in the northern region.

**Table 4:** Relative risk ratio (RRR) and their 95%CI estimated from the multinomial regression analysis for the type of tobacco use among females in India, GATS India, 2009–10.

	Smoking only		Smokeless tobacco useonly		Dual use of tobacco	
	RRR	95%CI	RRR	95%CI	RRR	95%CI
**Age group**						
15–24®	1.00		1.00		1.00	
25–44	12.57**	[5.11–30.88]	1.94**	[1.66–2.26]	2.23*	[1.07–4.67]
45–64	33.33**	[13.53–82.07]	3.44**	[2.91–4.06]	6.55**	[3.10–13.82]
65+	50.47**	[19.48–130.77]	4.47**	[3.67–5.45]	15.20**	[6.74–34.29]
**Residence**						
Urban®	1.00		1.00		1.00	
Rural	1.71*	[1.12–2.60]	1.13	[0.97–1.32]	1.58	[0.96–2.62]
**Education**						
More than secondary®	1.00		1.00		1.00	
Upto secondary	0.55	[0.14–2.19]	1.71**	[1.29–2.27]	0.69	[0.20–2.35]
Upto primary	3.70	[0.97–14.05]	3.38**	[2.57–4.44]	1.45	[0.45–4.61]
No education	13.52**	[4.10–44.51]	4.41**	[3.38–5.76]	3.33*	[1.03–10.69]
**Occupation**						
Govt./Non-Govt. employee®	1.00		1.00		1.00	
Self employed	0.54*	[0.31–0.94]	0.88	[0.74–1.06]	0.20**	[0.11–0.37]
Homemaker	0.68	[0.42–1.11]	0.65**	[0.56–0.76]	0.31**	[0.18–0.53]
Others^(a)^	1.09	[0.59–2.02]	0.70**	[0.57–0.87]	0.26**	[0.11–0.59]
**Household asset**						
Rich®	1.00		1.00		1.00	
Moderate	1.34	[0.84–2.13]	1.68**	[1.42–1.97]	1.88	[0.98–3.60]
Poor	1.54	[0.92–2.56]	2.04**	[1.71–2.42]	2.39*	[1.18–4.84]
**Smoking causes heart attack**						
Yes®	1.00		1.00		1.00	
No	1.72*	[1.07–2.76]	1.22**	[1.08–1.39]	0.81	[0.52–1.25]
**Smoking causes stroke**						
Yes®	1.00		1.00		1.00	
No	0.91	[0.59–1.39]	0.90	[0.80–1.03]	0.47**	[0.33–0.67]
**Smoking causes lung cancer**						
Yes®	1.00		1.00		1.00	
No	0.80	[0.51–1.27]	1.08	[0.91–1.28]	2.18**	[1.34–3.55]
**Smokeless tobacco use** **causes serious illness**						
Yes®	1.00		1.00		1.00	
No	1.37	[0.88–2.15]	0.95	[0.79–1.14]	1.31	[0.74–2.30]
**Region**						
North®	1.00		1.00		1.00	
Central	0.93	[0.60–1.43]	14.11**	[9.79–20.34]	4.01**	[2.20–7.31]
East	1.37	[0.91–2.07]	30.30*	[20.77–44.18]	3.51**	[1.56–7.87]
North-east	1.58*	[1.08–2.31]	41.45**	[28.02–61.32]	20.10**	[9.67–41.79]
West	0.06**	[0.02–0.16]	14.81**	[10.14–21.65]	1.38	[0.49–3.87]
South	0.66	[0.41–1.04]	10.53**	[7.01–15.82]	1.29	[0.42–3.89]

Note: CI-Confidence interval; *p<0.05, **p<0.01; ® = Reference category; Govt. = Government; ^(a)^Others includes student, retired or unemployed.

## Discussion

GATS-India data provides an ample opportunity to study the tobacco use behaviour in the form of smoking, smokeless tobacco and dual use of tobacco among adults in India. Understanding the type of tobacco use behaviour has important health implications. The estimates of the tobacco use provided by GATS are considered to be reliable and representative enough as the survey was designed to provide only tobacco use indicators in India. The other studies suffer from the most important limitation that they were not designed to collect information on tobacco use. Surrogate responses were used which often produced bias and inaccuracies in the results [5]. This study comprehensively investigates the socio-economic, demographic and knowledge-related factors associated with type of tobacco use in India.

Tobacco consumption among females was mostly in the form of smokeless tobacco, while that among males was distributed among the three types of uses. Tobacco use in India has been higher among males than among females in India. However, the male-female gap was lower in the case of use of smokeless tobacco. The use of smokeless tobacco was equally high among middle-aged and elderly males and females. This indicates that adult females in India were as vulnerable as males, and at a high risk of using smokeless tobacco, especially in the higher age groups. With an increase in age, the odds of using tobacco significantly increase in India. Elderly and middle-aged females had significantly higher odds of consuming every type of tobacco. Smoking and dual use of tobacco among males were more common in the younger and middle-aged adults. Age has been found to be an important determinant of tobacco use in earlier studies [1], [5]. Tobacco use was found more common among the uneducated people in the country. Education was one of the most important determinants of tobacco use irrespective of the type of use. Uneducated males and females in India were at a higher risk of using tobacco. This can often be attributed to less knowledge and awareness among the uneducated people. Being poor was significantly associated with a higher risk of use of smokeless tobacco among males, and use of smokeless tobacco and dual use of tobacco among females in India. The relation between these socioeconomic markers and tobacco consumption is similar to relations observed in developed countries and other studies done in previous decades in India [1], [13]. Given their high degree of fatalism, poor people in India are more likely to initiate tobacco use early. The outcomes also indicate that the tobacco use was relatively lower among males and females in the unorganized sector, such as the self-employed, than among the people in the organized sector, that is, government and non-government employees. This certainly necessitates the importance of approaching this population and motivating them to stop tobacco use.

Tobacco use in India varies significantly by states and regions. A comparison of state-wise figures for smoking and smokeless tobacco use among adults aged 15 years and above, provided by another research study for India, [1] with figures provided by GATS-India offer some useful insights into the change in the prevalence of tobacco use from 1998–99 to 2009–10 in the country. The comparison shows that tobacco use in India has increased in many states. More than two-third states of India are experiencing an increase in the prevalence of smokeless tobacco use both among males and females. Approximately half of the states in India are experiencing an increase in smoking among females. These increasing figures are clearly an alert for the government to stringently implement tobacco control programmes. Region was one of the significant determinants of smokeless tobacco use and dual use of tobacco in India. Males in the north-east had 4 times and females 41 times higher RRR to use smokeless tobacco. There are other studies which show that the regional variations in tobacco use have been significant in the earlier decades too [1]. This study shows that adults in the north-east region are among the most vulnerable population subgroups in India. This study also shows that, in India, the non-awareness of health hazards increases the likelihood of using tobacco. It significantly affects smoking among males. The risk of consuming other forms of tobacco use was also significantly affected by the non-awareness of the selected health hazards of tobacco use. Other studies suggest that the knowledge of health hazards of tobacco is significantly related to avoidance behaviour. The severity of health risks is though sometimes not adequately understood by tobacco users [14]. To that extent, there is a need to spread comprehensive information about the health hazards of tobacco use among every subsection of the society. Cancer is known among the masses as the most common disease induced by smoking. There are numerous other non-communicable diseases, such as stroke and heart attack, which are caused by smoking. The awareness about this latter correlation was found to be lower as compared to that about cancer. The GATS-India report shows that the knowledge of health hazards of smoking and smokeless tobacco use is high in India, yet there is a need to increase this knowledge with more efficiency [11].

## Conclusions

Tobacco use in India is clearly a big burden in terms of its magnitude and use in different forms. There is also a need to check the dual use of tobacco in India. Dual users are at much higher health risks than those who consume the individual tobacco product. The higher prevalence of tobacco use among males, the uneducated and the poor is a matter of concern as these people even lack resources to combat the morbidity associated with tobacco use. At a time when India is experiencing a major mortality and epidemiological transition, these subgroups are likely to suffer more from the dual burden of communicable and non-communicable diseases. In addition to the differences in the prevalence of tobacco consumption between disadvantaged and better off groups, the type and amount of tobacco consumption, and its dependency may also vary between these groups, further aggravating the differences in the disease burden attributable to tobacco. GATS report shows that more than 60 percent tobacco users consume first tobacco of the day within half an hour of waking up. An ample proportion of daily smoker consumes more than 10 cigarettes or *beedis* per day. Such figures were much higher for the uneducated and the male population [11]. From the tobacco control programme point of view, there is a need to monitor socioeconomic and state level inequalities in the tobacco use in India. The states which need more focus from the tobacco control programme point of persuasion are the north-eastern states, where an increasing trend in tobacco consumption has been observed over a period of time. Mass media campaigns in tobacco control programmes require spotlighting an array of diseases that are induced by smoking and smokeless tobacco use. The tobacco control programme was piloted in India in 2007–08 and started in 42 districts in the country [12]. The coverage of this programme needs to be increased to more districts in states with a higher prevalence. There may be several reasons which we can relate for such a high burden of use of tobacco in India. Marketing efforts of the tobacco industry, targeting of the young people, weak enforcement of tobacco control policies, continuing affordability of tobacco products, and inadequate knowledge about the harmful effects of tobacco are all factors contributing to a high use of tobacco [15].
